# Contact, cognition, and taiwanese youths’ attitudes toward local and Chinese culture

**DOI:** 10.1371/journal.pone.0344614

**Published:** 2026-03-27

**Authors:** Yafeng Xu, Liliang You

**Affiliations:** 1 School of Economics and Management, Suqian University, Suqian City, Jiangsu, China; 2 Graduate Institute of Development Studies, Taiwan Chengchi University, Taipei City, Taiwan, China; Cardiff University, UNITED KINGDOM OF GREAT BRITAIN AND NORTHERN IRELAND

## Abstract

Classical contact theory posits that intergroup contact can reduce perceived threats and hostility between in-groups and out-groups, thereby laying the foundation for the formation of subsequent friendships and deeper forms of identification. However, in a cross-Strait context that is highly politicized and in which exchanges are deliberately obstructed by the Democratic Progressive Party, whether contact experience alone is sufficient to trigger changes in Taiwanese youths’ attitudes toward Chinese culture remains an open question requiring further examination. Drawing on data from the 2018 Taiwan Social Change Survey, this study analyzes a sample of youths aged 18–45 and constructs an index of attitudes toward “Taiwan’s local culture” to examine the effects of two independent variables, namely Taiwanese youths’ contact with the Chinese mainland and increases in Taiwanese youths’ cognition of the Chinese mainland, on their cultural attitudes. The results show that experience of contact with the Chinese mainland alone does not have a statistically significant effect on attitudes toward local culture. By contrast, individuals with higher levels of cognition of the Chinese mainland exhibit a significantly lower Taiwan’s Local Cultural Attitude Index. These findings suggest that under conditions in which cross-Strait exchanges are constrained, contact per se may be insufficient to induce shifts in cultural attitudes. Instead, its effects are more likely to operate through increases in cognition.

## Introduction

Contact theory suggests that intergroup contact can enhance cultural understanding, and increase mutual understanding between in-groups and out-groups, thereby reducing prejudice [1,2]. However, existing studies have focused primarily on how contact reduces prejudice or increases group attitudes [3]. As a result, relatively limited attention has been paid to how intergroup contact shapes more complex and deeper cultural attitudes. In particular, much of the literature emphasizes whether contact occurs and how frequently it takes place, while offering little systematic analysis of which factors are most strongly associated with the translation of contact experiences into supportive attitudes. More recent research has increasingly recognized the importance of cognition in the effects of intergroup contact [4]. Especially in highly diverse and politically polarized social contexts, cognitive factors appear to play a critical role in the complex process through which group attitudes are formed. For example, in the absence of direct contact with out-groups, individuals’ cognition of out-groups often relies on indirect information and preexisting narrative frameworks, which may lead to misjudgments in their attitudes toward out-groups. Correspondingly, once individuals engage in actual contact with out-groups, changes in their cognitive understanding of out-groups may further prompt reflection on and revision of their existing cultural attitudes toward in-groups. Indeed, interactions and exchanges between cross-Strait youths offer important insights into contact theory from the perspective of cultural attitudes.

In recent years, although the Democratic Progressive Party has continued to restrict and impede normal interactions across the cross-Strait through a series of policy measures, the Chinese mainland has actively promoted the normalization and routinization of cross-Strait exchanges, with cultural interactions between the two sides remaining vibrant. On the one hand, in 2024, cross-strait travel reached 4.405 million person-times, representing a year-on-year increase of 53.8%; among these, visits from Taiwanese to the Chinese mainland numbered 4.023 million, up 54.3% from the previous year [5]. On the other hand, compared with earlier periods of direct cross-Strait public exchanges, the current forms of contact show a trend toward diversification, encompassing not only traditional face-to-face interactions but also virtual interactions through games, films, and social media. Against this backdrop, virtual contact via the Internet and new media has gradually become an important channel for Taiwanese youths to learn about the Chinese mainland and Chinese culture. Under these circumstances, theoretical reasoning would suggest that frequent exchanges should help alleviate Taiwanese youths’ prejudices toward the Chinese mainland and have a positive impact on their attitudes toward Chinese culture. Nevertheless, empirical evidence indicates that such identity has not strengthened with the increase in exchange frequency; rather, it has declined, accompanied by a stronger attachment to what is framed as Taiwan’s “local culture”. It should be emphasized that, under the Democratic Progressive Party’s sustained narrative, Taiwan’s “local culture” has been reconstructed as distinct from, and even in opposition to, Chinese culture, carrying the implication that Taiwan does not share common cultural roots with the Chinese mainland. Survey data further reveal that between 2013 and 2023, the proportion of Taiwanese youths who agreed that “Taiwanese culture has diverged from Chinese culture” rose from 52.0% to 67.7% [6].

It should be noted that cultural attitudes and cultural identity are two related but distinct concepts. Cultural attitude, which can also be understood as cultural acceptance, primarily refers to individuals’ evaluative orientations toward and levels of acceptance of specific cultural objects. Cultural identity, by contrast, involves the internalization of culture as part of self-conception and group belonging. Logically, cultural acceptance constitutes one of the important preconditions for the formation of cultural identity, but it does not necessarily lead to the emergence of identity. Compared with cultural identity, cultural attitudes places greater emphasis on individuals’ perceptions and expressions regarding their own culture and that of others. As such, it is more conducive to clear conceptual operationalization in empirical research, thereby avoiding the over-inference of attitudinal differences as changes at the level of identity or identification.

From a cultural origin perspective, Taiwan and the Chinese mainland both belong to the broader Chinese cultural system and share common historical trajectories and cultural foundations. However, under the long-term influence of political and ideological confrontation across the cross-Strait, cultural discourse within Taiwan has been shaped by partisan politics and has gradually shifted. Specifically, after the Democratic Progressive Party assumed power in the early 2000s, it implemented “de-Sinicization” measures and emphasized the uniqueness of “Taiwan’s local culture”. In this process, Chinese culture has gradually been marginalized and, in some social groups within Taiwan, even regarded as an “other culture” distinct from Taiwanese culture. This has resulted in a clear fragmentation and polarization of overall cultural attitudes within Taiwan society.

It is precisely within this highly politicized and culturally polarized social context that examining the effects of Taiwanese youths’ contact with the Chinese mainland and their perceived increase in cognition of the Chinese mainland on their cultural attitudes toward in-groups and out-groups becomes a question of both theoretical and practical significance. Therefore, this study draws on data from the 2018 Taiwan Social Change Survey and employs multiple linear regression models to empirically analyze a sample of Taiwanese youths aged 18–45. It investigates whether contact with the Chinese mainland and changes in cognition of the Chinese mainland affect Taiwanese youths’ cultural attitudes toward in-groups and out-groups under conditions of political tension and constrained cross-Strait exchanges, thereby empirically enriching the depth and scope of applications of intergroup contact theory.

## Literature review

### The cultural context and historical trajectory of cross-Strait contact

Cultural identity in Taiwan society has exhibited a dynamic and pluralistic character amid long-term political and social transformations. After the Chinese Kuomintang (KMT) retreated to Taiwan in 1949, Chiang Kai-shek’s regime, driven by the political necessity of “opposing the Communist Party of China and reviving the Republic of China”, emphasized Taiwan as the representative of “Free China”. Through education and cultural policies, Taiwan authorities promoted Mandarin instruction and reinforced traditional Chinese culture in order to sustain Taiwanese residents’ identity as Chinese and to preserve cultural ties with the Chinese mainland [7,8]. During this period, under conditions of long-term cross-Strait isolation and the near suspension of interpersonal exchanges, social identity and cultural identity became highly overlapping, resulting in a closed pattern characterized by low contact but high cultural identity.

After the lifting of martial law in 1987, Taiwan’s democratization process accelerated political and social transformations. The second generation of Chinese Mainlanders (waishengren), who were born in Taiwan to families with ancestral origins in the Chinese mainland, grew up without direct memories of the Chinese mainland. As a result, the mobilization slogan of retaking the Chinese mainland gradually lost its practical plausibility. At the same time, under the pressures of democratization, the Kuomintang progressively moved toward “Taiwanization” [9]. After Lee Teng-hui assumed office, he introduced the concept of the “New Taiwanese”, which emphasized ethnic integration and identity with Taiwan as the core, seeking to mitigate ethnic divisions and promote localization policies. During this stage, cultural identity in Taiwan society began to shift from being centered solely on Chinese culture to a more pluralistic model in which Taiwanese local culture and Chinese culture coexisted. Although cross-Strait family visits and economic exchanges resumed during this period, contact remained limited in scope and was still at an embryonic stage.

Entering the 21st century, after Chen Shui-bian came to power, the policy of “de-Sinicization” was further intensified. From the renaming of cultural symbols and the revision of textbooks to the implementation of minority policies, Chinese culture was gradually marginalized and placed within a framework positioned in opposition to Taiwan’s local culture [10]. During this period, “Taiwan’s cultural identity” gradually shifted toward a local orientation, accompanied by political democratization, resulting in a dual transformation at both the institutional and cultural levels. Although cross-Strait economic interactions steadily increased, political relations remained tense and institutionalized exchanges were constrained, producing a paradoxical situation in which political barriers coexisted with economic exchanges.

After Ma Ying-jeou assumed office in 2008, cross-Strait relations entered a peak period of contact. Under the principle of the “1992 Consensus”, cross-Strait significantly relaxed restrictions on tourism, education, and economic exchanges. The number of visitors from the Chinese mainland to Taiwan increased from fewer than 100,000 in 2008 to 3.3 million in 2014 [11]. A large number of Taiwanese business people and students moved to the Chinese mainland to pursue development opportunities, which significantly increased occasions for social contact. Survey data indicate that in 2013, 65 percent of respondents reported casual encounters with Chinese mainland tourists in public places, and by the end of 2014 this proportion had risen to 83 percent. More than one-third of respondents had friends from the Chinese mainland, among whom approximately 16–22 percent maintained work-related relationships with Chinese mainland citizens [12]. However, the high frequency of contact did not result in the anticipated increase in cultural identity. Most people in Taiwan continued to resist defining themselves as Chinese, instead developing a dual identity as ethnically Chinese but politically Taiwanese [13].

However, during the administrations of Tsai Ing-wen and Lai Ching-te of the Democratic Progressive Party (DPP), cross-Strait exchanges were substantially curtailed. Taiwan authorities continued to expand the “restricted list” of exchanges and introduced alternative programs, such as the Youth Overseas Dream-Building Fund Plan, to redirect youth exchange activities toward regions other than the Chinese mainland. The Taiwanese public shows greater acceptance of a “local culture” orientation. Under the current situation in which direct contact is restricted due to obstruction by the Democratic Progressive Party, digital media has provided Taiwanese youths with a channel for virtual contact. A 2024 survey conducted by the Taiwan Public Opinion Foundation further revealed that among citizens aged 20 and above, identity as “Chinese only” increased by 3.5 percentage points compared with June of the same year and, unusually, surpassed the proportion identifying as both Taiwanese and Chinese. This shift reflects a growing diversification in young people’s attitudes toward Chinese culture and the image of China amid globalization and the Chinese mainland’s rise.

Based on the foregoing review of the historical context, the core concepts of “Chinese culture” and “Taiwan’s local culture” in this study are defined as follows. Chinese culture is a civilizational form of culture centered on the Chinese writing system, Confucian ethical order, and historical memory, and is characterized by continuity and inclusiveness across different institutional and geographical settings [14]. By contrast, although Taiwan’s local culture substantively falls within the category of Chinese local culture, since the 1990s it has been endowed with specific political connotations and consciously elevated as a core identity distinguishing Taiwan from China, thereby shifting from a form of local culture to a politicized narrative [13]. In this process, Taiwan’s local culture has ceased to function as a regional expression within the broader Chinese cultural system and has instead been constructed as a cultural symbol distinct from China and closely tied to Taiwanese identity. Accordingly, in this study, attitudes toward Chinese culture and attitudes toward Taiwan’s local culture are treated as two opposing cultures constructed under the Democratic Progressive Party’s discourse. Multiple studies have substantiated this pattern. For example, Hsieh Ta-ning’s research indicates that among individuals aged 18–30, approximately two-thirds no longer consider themselves part of the Chinese nation and no longer perceive themselves as belonging to the Chinese cultural sphere [15]. Based on survey data from “Academia Sinica” in Taiwan, Su Sung-hsing and colleagues find that only 16.1 percent of respondents develop an emotional connection on the basis of Chinese culture or the landscapes of the Chinese mainland. The underlying reason for this result is that Taiwanese youths take considerable pride in the achievements of contemporary Taiwanese culture, fashion, and creative industries [16]. In sum, whether viewed from the perspective of historical evolution or the current social reality, the local culture constructed by the Democratic Progressive Party in Taiwan has become oppositional to Chinese culture, with the two being artificially constructed as cultures of different groups.

However, Taiwan’s local culture has inherent endogenous limitations. Based on Clifford Geertz’s perspective, culture constitutes a web of meanings through which people understand and interpret their world [17]. Through culture, a nation or an ethnic group distinguishes itself from other nations or cultural communities. This cultural identity provides the foundation for collective identification and binds members into a coherent whole. Therefore, when promoting culture, any government should pay attention to cultural diversity and balance, and avoid an overly homogenized cultural policy.

In modern societies, the formation and transformation of culture are not necessarily the result of organic processes. The construction of Taiwan’s local culture, for instance, has been closely linked to the manipulation, narratives, and cultural policies of political elites, particularly a series of “de-Sinicization” policies promoted by the Democratic Progressive Party (DPP). These cultural policies generally exhibit a strong localist orientation and are often accompanied by explicit political agendas. Such policies tend toward cultural homogenization, insofar as they frequently conflate “Taiwan’s local culture” with cultural diversity more broadly, whether in education, the cultural and creative industries, or public activities.

The danger of politicizing culture lies in its neglect of the reality of cultural pluralism within Taiwan society. Rather than promoting cultural diversity, this approach may instead produce cultural ruptures and intensify tensions and antagonisms among different cultural groups. While the Taiwan authorities’ emphasis on local culture is, in essence, aimed at strengthening a local Taiwanese identity, this orientation has already brought qualitative changes to Taiwanese local cultural, social, and political landscapes. In particular, against the backdrop of increasingly tense cross-Strait relations, it has gradually taken on elements of a narrow form of nationalism.

### Social identity theory and contact theory

As noted above, in Taiwanese youths’ cultural cognition, Chinese culture is no longer regarded as part of their own cultural system but is instead categorized as an out-group. Under the premise that cross-Strait group boundaries have already been established, social identity theory and contact theory provide an analytical foundation for understanding changes in Taiwanese youths’ cultural attitudes. Social identity theory posits that individuals categorize themselves as members of particular social groups in order to obtain a positive social identity, thereby enhancing their self-esteem and sense of belonging [18,19]. In contexts where social categories become salient, individuals tend to construct their self-concept based on group prototypes and to conform to group norms in order to maintain group boundaries and internal cohesion [20]. Such identity is not static but dynamically shifts between personal and collective identities depending on contextual cues. When the external environment accentuates ethnic or cultural differences, the salience of group prototypes increases, prompting members to interpret social reality from a collective standpoint. For Taiwanese youth, the political issues of cross-Strait relations, media narratives, and generational experiences continuously activate the dual categories of Taiwan and Chinese, causing their self-positioning to oscillate between a sense of local identity and a recognition of Chinese cultural affiliation.

Building upon social identity theory, cultural identity theory further posits that symbolic systems such as language, customs, and values are internalized through everyday communication and socialization, and are continuously negotiated and reconstructed across different contexts of interaction [21,22]. From a psychological perspective, Yan further emphasizes that cultural identity derives its meaning through the differentiation between in-groups and out-groups in intercultural interactions, and is continuously renegotiated in response to contextual, relational, and psychological needs such as belonging, recognition, and consistency [23]. For Taiwanese youth growing up in the context of globalization and the rise of Chinese mainland, the negotiation of cultural identity involves not only value judgments between “Taiwanese local” and Chinese cultures, but is also influenced by cognitive understandings of Chinese mainland society, economy, and culture. In other words, changes in cultural identity are driven by multiple factors, including cognition, and cannot be fully explained by political stance alone.

Identity theory and cultural identity theory highlight the negotiated outcomes of identity across different interactional contexts. Intergroup contact theory, however, supplements this perspective by specifying the pathways through which identities can change. Pettigrew points out that, under positive conditions such as equal status, cooperative goals, and institutional support, intergroup contact can reduce stereotypes and enhance intergroup understanding. Pettigrew argues that under favorable conditions such as equal status, cooperative goals, and institutional support, intergroup contact helps reduce stereotypes and enhance mutual understanding between groups [1,2]. Subsequent research has further shown that the effects of contact operate by increasing understanding of the outgroup, thereby reducing intergroup anxiety and ultimately facilitating recategorization. At the same time, factors such as the duration of contact, the intensity of interaction, and the degree of emotional involvement all shape the outcomes of contact [24]. However, in highly politicized societies, individuals often develop defensive cognitions aimed at protecting their personal identities and tend to favor their ingroup in order to maintain a sense of ingroup superiority [25], which may render contact ineffective. Even when conditions of equal status contact are formally met, individuals’ understanding and evaluation of outgroups may still be strongly influenced by political discourse and media framing [26]. With broader social changes, scholars have further developed theories of indirect contact, including “extended contact”, “vicarious contact” and “imagined contact” [27]. A large body of research demonstrates that indirect contact through information conveyed by friends or through media exposure can indeed increase attitudes toward outgroups [28]. Taken together, these developments extend the scope of contact theory beyond face-to-face interaction to include mediated and psychological dimensions.

At the same time, threat theory suggests that when contact situations elicit symbolic or realistic threats, the positive effects of contact may be attenuated or even reversed [29,30]. Even in the absence of objective grounds, individuals’ subjective perceptions of threat can significantly exacerbate intergroup conflict and prejudice. For Taiwanese youth, if contact elicits perceived threats related to political system differences, security, or cultural value conflicts, frequent interaction may nonetheless reinforce local consciousness or trigger defensive forms of identity. This potential threat effect is particularly pronounced during periods of Democratic Progressive Party rule, when cross-Strait exchanges were restricted and media narratives tended to “otherize” the Chinese mainland, further undermining the positive effects of contact.

In summary, social identity theory and cultural identity theory elucidate the logic underlying the formation of youths’ cultural identity, while intergroup contact theory and threat theory clarify the conditions and constraints under which contact leads to attitudinal change. Intergroup contact theory, social identity theory, and threat theory all suggest that the effects of contact on attitudes are not linear or uniform. Rather, the impact of contact is highly contingent on multiple factors, including group identification, frequency and duration of contact, the salience and framing of contact, perceived threat, the political environment, and the medium through which contact occurs. It should be noted, as emphasized earlier, that cultural identity represents a deeper level of attitude than cultural acceptance. Factors capable of influencing cultural identity should therefore also have effects on levels of cultural acceptance. However, existing theoretical research has largely remained focused on verifying the role of contact in reducing prejudice and still lacks systematic examination of how contact brings about attitudinal transformation.

### Research on cross-Strait contact and identity

In recent years, studies on cross-Strait contact and identity have shifted from early efforts that merely sought to verify the existence of contact effects to more nuanced investigations of the types and quality of contact, as well as their differential impacts on various dimensions of identity. Overall evidence indicates that contact does not uniformly produce positive outcomes; its effects are highly contingent on the nature, target, and context of interaction, and may vary across different attitudinal levels. This trend not only responds to Pettigrew’s classical propositions on contact theory, but also highlights how the unique political sensitivities of the Taiwan Strait shape the outcomes of cross-Strait contact. Existing studies likewise indicate that interactions that remain superficial are insufficient to induce fundamental attitudinal change. Instead, increases in cognition of out-groups following contact constitute the most critical factor in altering attitudes toward out-groups [31].

Firstly, in quantitative research, scholars generally find that the type of contact determines both the direction and strength of its effects. Wang and Cheng, distinguishing between “casual contact” and “meaningful contact”, note that incidental interactions between Taiwanese residents and Chinese mainland tourists in settings such as shopping malls, parks, or restaurants do not significantly alter overall evaluations of the Chinese mainland. In contrast, more in-depth interactions established through friendships can mitigate negative impressions of Chinese mainland individuals, yet still struggle to substantially shift attitudes toward the Chinese mainland as a whole. This finding aligns with the classical expectations of contact theory, which suggest that low-quality or brief interactions are unlikely to leave lasting impressions on individual cognition, whereas sustained and high-quality interactions are necessary to produce prejudice-reducing effects. Meng, using a structural equation modeling approach, found that Taiwanese individuals who had direct contact with Chinese mainland exhibited significantly reduced negative stereotypes toward the Chinese mainland, indicating that contact can operate by mitigating prejudice [32]. Based on the 2015 “Cross-Strait Relations and Security” survey, Kuan employed cross-tabulation and ordinal logistic regression models to show that even when Taiwanese youth are immersed in high-frequency economic and trade exchanges over the long term, they find it difficult to develop deep identity with the Chinese mainland, yet continue to support the maintenance of cross-Strait economic interactions, reflecting a contradictory orientation [33]. These findings indicate that even when exposed to long-term, high-frequency contact, Taiwanese youth continue to exhibit a contradictory orientation in their political identity.

Secondly, qualitative and mixed-method studies offer more nuanced contextual insights into the dynamics of intergroup contact. Drawing on content analysis, Yi-wei Huang found that cross-Strait youth exchanges have substantially strengthened mutual emotional bonds and increased the quality of interaction [34]. Similarly, Ya-feng Xu, based on in-depth interviews, found that while Taiwanese youth tend to hold an open attitude toward Chinese culture, insufficient cognitive understanding constrains the formation of deeper cultural identity [35]. Consistent results were found in the study by Shu Keng and Jean Yu-chen Tseng, which indicated that while short-term visits to the Chinese mainland tend to mitigate stereotypical perceptions, they fail to produce meaningful shifts in political orientation [36]. Notably, Hongjuan Shang and Yiping Zhang further pointed out that when intergroup contact triggers identity threat or security anxiety, such contact may not foster identity integration but instead reinforce Taiwanese youths’ sense of “local consciousness” and political defensiveness [37]. These studies suggest that the effects of intergroup contact are not linear but highly context-dependent. When perceived threats are activated, they may offset or even reverse the positive outcomes of contact. Further experimental evidence has identified potential mechanisms for mitigating intergroup hostility. Chiang, through situational experiments and agent-based simulations, confirmed the effect of “indirect reciprocity”, showing that when outgroup members act benevolently toward ingroup members, individuals tend to respond with positive reciprocity, thereby contributing to the long-term increase and stabilization of cross-Strait intergroup relations [38].

In sum, existing studies provide two important insights for the research design of this article. First, prior research points to a relatively consistent empirical finding that Taiwanese youths may experience attitudinal change toward the Chinese mainland following cross-group contact. Building on this research trajectory, this study adopts “whether the respondent has visited the Chinese mainland” as a key indicator of Taiwanese youths’ direct cross-group contact. This operationalization has clear theoretical grounding and practical validity for analyzing its potential effects on cultural attitudes. Second, with regard to the substantive focus of existing literature, attitudinal changes among Taiwanese youths resulting from contact with the Chinese mainland have primarily been examined in relation to political or instrumental issues such as positions on unification and independence, economic and trade attitudes, and willingness to engage in exchanges. By contrast, empirical research on cultural attitudes, a dimension more closely related to values and identity, remains relatively limited and has yet to develop a systematic analytical framework. In light of this gap, this study extends its analytical focus to the domain of cultural attitudes. It specifically examines the effects of Taiwanese youths’ contact experiences with the Chinese mainland and their increased cognition of the Chinese mainland on their attitudinal orientations toward Chinese culture and local culture, thereby addressing the relative neglect of the cultural dimension in existing research.

## Materials & methods

### Sample selection and description

This study uses data from the 2018 “Globalization and Culture” module of the Taiwan Social Change Survey (TSCS), conducted by the Institute of Sociology, “Academia Sinica”. The TSCS, launched in 1984, is the longest-running and most comprehensive nationwide social survey in Taiwan, covering multiple disciplines. The survey is conducted every five years with rotating thematic modules and is aligned with the International Social Survey Programme (ISSP). The 2018 survey employed a stratified multistage probability proportional to size (PPS) sampling design, using household registration records as the sampling frame. Sample weights were provided to ensure representativeness with respect to gender, age, and regional distribution. In line with cross-Strait policies and existing scholarly research, which commonly define “youth” as individuals aged 18–45, this study selects respondents aged 18–45 in Taiwan as the analytical sample [39,40]. After excluding cases with missing values on key variables, the final analytical sample consisted of 920 respondents. Analyses were conducted using the sample weights provided by “Academia Sinica” to enhance the generalizability and statistical precision of the results. It should be noted that the subsequent model estimation and statistical analysis were conducted using IBM SPSS Statistics 27 software.

### Variables and data processing

#### Dependent variable: Cultural attitude.

According to Schwartz, the cultural values of a society can be assessed through the aggregation of individual value orientations [41]. Therefore, this study draws on relevant questions from the multiculturalism module of the Taiwan Social Change Survey to construct two indicators reflecting the cultural identity attitudes of Taiwanese youth, based on attitudinal observation.

The first indicator is the Chinese cultural identity attitude index, which is constructed using three items from the questionnaire: “Chinese traditional culture should constitute an important part of Taiwan’s culture”, “We should strive to promote traditional Chinese culture”, and “Taiwanese culture is a continuation of traditional Chinese culture”. These items measure respondents’ attitudes toward the importance, continuity, and necessity of promoting Chinese culture.

The second indicator is the Taiwanese “local culture” identity attitude index. Given that the Democratic Progressive Party has constructed “Taiwan’s local culture” as an imagined, Westernized, and inauthentic culture, this index includes three items: “It is more important to learn the culture of advanced countries than to learn traditional Chinese culture”, “Taiwan should establish cultural characteristics different from those of the Chinese mainland”, and “Every Taiwanese should first understand their own culture”. These items assess respondents’ attitudes toward a “local culture” orientation. In the context and wording of the TSCS questionnaire, it implicitly suggests that Taiwanese local culture is portrayed as distinct from, and mutually exclusive with, Chinese culture.

It is worth noting that this study follows the original questionnaire’s seven-point Likert scale coding (1 = strongly agree, 7 = strongly disagree) without reverse scoring. The scores of the three items for each indicator were summed to form the “Chinese Cultural Attitude Index” and the “Taiwan’s Local Cultural Attitude Index” (hereafter referred to as Local Culture Attitude Index), respectively. Therefore, the lower the scores on these two indicators, the higher the respondents’ acceptance of the corresponding cultural orientation, whereas higher scores indicate lower acceptance.

#### Independent variables: contact variable and cognition variable.

Intergroup contact theory suggests that once individuals have formed social identities, cross-group contact can reduce their prejudice toward out-groups [42]. The specific mechanism operates by enabling individuals, through intergroup contact, to recognize the diversity within out-groups, thereby weakening established stereotypes, and by fostering emotional connections that help alleviate hostility and conflict [2]. Therefore, after individuals come into contact with out-groups, the process also involves the formation of understanding and increased cognition of out-groups during contact. In light of the real-world context in which the research subjects are situated, this study defines two core independent variables, namely a “contact variable” and a “cognition variable”.

Contact variable: Whether the respondent has visited the Chinese Mainland. Face-to-face interaction is regarded as the most direct and effective context for communication between out-groups and in-groups. A meta-analysis by Pettigrew and Tropp, integrating 515 studies, shows a significant negative correlation between direct contact and prejudice, and this effect demonstrates robustness across contexts [24]. On this basis, this study adopts the survey item “whether the respondent has visited the Chinese mainland (excluding Hong Kong and Macao)” as the first independent variable, namely the contact variable. In the data, this variable is dichotomized, with respondents who have visited the Chinese mainland coded as 1 and those who have not coded as 0.

Cognition Variable: The respondent’s perceived increase in cognition of the Chinese mainland. A review of the literature indicates that contact does not necessarily lead to understanding of out-groups. During the process of contact, individuals must also develop an adequate understanding of out-groups’ values and beliefs. Accordingly, changes in cognition of out-groups constitute a variable of particular interest. For this reason, the second independent variable in this study is the degree of change in Taiwanese youths’ cognition of the Chinese mainland. It should be noted that, due to limitations of the available data, it is not possible to measure in detail the specific content of respondents’ objective cognitive changes regarding the Chinese mainland. Moreover, given current global developments and the reality of constrained cross-Strait exchanges, alternative forms of contact such as online contact and other forms of vicarious contact have expanded to become the most important means through which Taiwanese youths engage with the Chinese mainland [43]. In operationalizing this variables, this study does not measure Taiwanese youths’ level of objective cognition of the Chinese mainland.

Instead, it focuses on the subjective changes in their cognition of the Chinese mainland as perceived through online contact. Based on these considerations, this study quantifies cognition using the survey question, “through internet use, which of the following countries or regions has your understanding increased the most?”. It should be noted that this indicator does not reflect individuals’ absolute level of cognition but rather a cognitive increment, that is, whether individuals perceive an increase in their understanding of the Chinese mainland as a result of Internet use. For respondents who already possessed a relatively high level of cognition of the Chinese mainland, Internet use may not have further enhanced their understanding and thus appears as a lower value on this indicator. It is precisely this variation that provides the basis for explaining cognitive change in the statistical analysis. In terms of operationalization, if respondents listed the Chinese mainland in any of the ranked positions (first, second, or third), they are considered to have acquired symbolic cognition of the Chinese mainland through the Internet. The three ranked positions are assigned values of 3, 2, and 1, respectively, while those who did not mention it are assigned a value of 0. Higher scores on this variable indicate a greater perceived increase in cognition of the Chinese mainland through online contact.

#### Control variables: Gender, age, and education.

To account for the potential influence of demographic differences on the measurement of cultural attitude, this study includes three control variables in the model: gender, age, and educational attainment. The rationale is as follows.

First, there are significant differences in identity and cultural identification across biological genders. Some studies indicate that in Taiwan, women tend to exhibit weaker orientations than men on indicators related to nationalism or national identity, suggesting that gender differences may entail systematic variations in the formation of identity [44]. Therefore, gender differences may also influence attitudes toward local culture [45]. In this study, the gender variable from the original survey was dichotomized, with females coded as 0 and males coded as 1.

Second, age is a key dimension in generational differences. Scholars generally hold that, compared with older cohorts, younger generations are more inclined to embrace local culture and identity [46]. Here, age is included as a continuous variable in the regression model to capture gradual differences in cultural attitudes across age groups. Given that the survey data are based on 2018, this study measures age using the “century age” method, calculated by subtracting the respondent’s year of birth from 2018, thereby obtaining a standardized and comparable age variable.

Finally, educational attainment serves not only as an indicator of socioeconomic status but is also related to the content of curricula that convey exclusive nationalist narratives. In recent years, Taiwan’s curricula and textbooks have contained substantial “de-Sinicization” narratives, which tend to reinforce exclusive attitudes among students. Consequently, cultural attitudes may vary across Taiwanese individuals with different levels of education [47]. Accordingly, educational attainment is coded as years of schooling, with high school coded as 12 years, university as 16 years, and so on, and treated as a continuous control variable. During data processing, any cases with missing or invalid codes for gender, age, or education (codes 92–99) were excluded to improve sample quality and ensure the reliability and external validity of the model results. The research variables and coding scheme used in this study are presented in Table 1.

**Table 1:** Research Variables and Coding Scheme.

Variable Types	Name	Item Code (Core Meaning)	Coding and Processing
Dependent Variables	Chinese Cultural Attitude Index (Y_1_)	F13e (Importance)	Sum of the original seven-point Likert scale scores. Strongly agree = 1, strongly disagree = 7. Total score of three items; lower scores indicate stronger support.
		F13f (Should Promote)	
		F13g (Continuity)	
	Taiwan’s Local Cultural Attitude Index (Y_2_)	F13b (Priority of Western Culture)	
Independent Variables	Contact Variable (X_1_)	B13a (Have been to the Chinese Mainland)	Dichotomized: Yes = 1, No = 0.
	Cognitive Variable (X_2_)	G9a, G9b, G9c (Countries or Regions Most Learned About Through the Internet)	If “Chinese mainland” is listed: 1st rank = 3, 2nd = 2, 3rd = 1; not listed = 0.
Control Variables	Gender	A1	Dichotomized: Male = 1, Female = 0.
	Age	A2	Age = 2018 − (1911 + A2).
	Education	A11	Mapped to continuous years of schooling, ranging from 6 to 21 years.

### Regression models

To examine the impact of Taiwanese youths’ contact with the Chinese mainland and changes in their cognition on cultural attitudes, this study employs ordinary least squares (OLS) multiple linear regression analysis, based on three main considerations.

First, regarding the measurement characteristics of the dependent variable, the Chinese culture and “local culture” indices in this study are constructed by summing multiple Likert-scale items, resulting in continuous variables. In empirical social science research, the use of OLS regression for such variables is widely accepted both theoretically and methodologically and aligns with standard practices in related studies. Second, in terms of research objectives, this study focuses on explaining the direction and relative influence of changes in independent variables on the dependent variable rather than predicting individual attitude scores. OLS regression allows for a clear presentation of the marginal effects of each explanatory variable while controlling for other factors. Third, from the perspective of model interpretability and comparability, OLS coefficients are straightforward to interpret and facilitate comparisons and extensions across different model specifications. Based on these considerations, the model constructed in this study is as follows:


$$Y_{i}=\alpha_{\text{0}}+\beta_{\text{1}}{Contact}_{i}+\beta_{\text{2}}{Cognition}_{i}+\beta_{\text{3}}{Contrils}_{i}+\mu_{i}$$

Where the dependent variable represents the Chinese culture or Taiwan’s Local Cultural Attitude Index of respondent *i*. The core independent variable Contact indicates whether the respondent has visited the Chinese mainland. The core independent variable Cognition denotes the respondent’s perceived increase in cognition of the Chinese mainland through online contact. Controls is a vector of control variables, including individual characteristics such as gender, age, and years of education.

## Results

### Descriptive analysis

The effective sample size of this study is 920 respondents. Overall, the sample exhibits a certain degree of variation in both the core variables and demographic characteristics, providing a solid data foundation for the subsequent empirical analysis.

With respect to the dependent variables, the Chinese Cultural Attitude Index ranges from 3 to 21, with a sample mean of 10.78 and a standard deviation of 4.12. This indicates that Taiwanese youth, on average, hold a moderate level of acceptance toward Chinese culture, while substantial variation exists across individuals, suggesting that the index possesses good discriminative capacity and analytical value. Meanwhile, the Taiwan’s Local Cultural Attitude Index ranges from 3 to 18, with a mean of 9.81 and a standard deviation of 2.60, indicating that the sample likewise exhibits a certain degree of individual variation in attitudes toward local culture. Based on the descriptive statistics, although both Chinese cultural attitudes and local cultural attitudes fall within a moderate range in terms of their mean values, they differ markedly in their levels of dispersion. The standard deviation of Chinese cultural attitudes is substantially higher than that of local cultural attitudes, implying that Taiwanese youth display greater polarization in their stances on issues related to Chinese culture, whereas attitudes toward local culture are characterized by higher consistency and stability. This contrast suggests that the two indices capture qualitatively different structures of cultural attitudes at the empirical level, thereby providing a sound basis for examining their respective determinants in subsequent analyses.

With respect to the independent variables, the first independent variable (X_1_), “whether the respondent has visited the Chinese mainland,” is a binary variable with a mean of 0.33 and a standard deviation of 0.47, indicating that only about one-third of the respondents have had direct experience of visiting the Chinese mainland. The second independent variable (X_2_), “the respondent’s perceived increase in cognition of the Chinese mainland,” ranges from 0 to 3, with a mean of 0.79 and a standard deviation of 0.98. This suggests that the overall increase in cognitive performance is relatively low, while substantial variation exists across individuals. Regarding the control variables, the gender distribution in the sample is largely balanced, with males accounting for approximately 50 percent. Respondents’ ages range from 19 to 45 years, with a mean age of 32.24 and a standard deviation of 7.851, indicating that the sample covers different stages within the youth cohort. Years of education range from 6 to 21, with an average of 14.33 years and a standard deviation of 2.424, suggesting that most respondents have attained at least a high school education or above, and that the overall educational level of the sample is relatively high.

Overall, the sample exhibits considerable individual variation across key variables, including local cultural attitudes, contact experiences, and cognitive levels, while maintaining a reasonable demographic structure. This provides a solid empirical basis for subsequent correlation analyses and regression models examining the effects of contact and cognition on Taiwanese youths’ attitudes toward local culture. The descriptive statistics of the core variables are presented in Table 2.

**Table 2:** Descriptive Statistics of Core Variables.

Variables	N	Minimum	Maximum	Arithematic Mean	Standard Deviation
Chinese Cultural Attitude Index (Y_1_)	920	3	21	10.78	4.12
Taiwan’s Local Cultural Attitude Index (Y_2_)	920	3	18	9.81	2.599
Contact Variable (X_1_)	920	0	1	0.33	0.47
Cognitive Variable (X_2_)	920	0	3	0.79	0.98
Male	920	0	1	0.51	0.5
Age	920	19	45	32.24	7.851
Years of Education	920	6	21	14.33	2.424
Valid samples	920				

#### Correlation analysis.

To preliminarily examine the hypotheses proposed above, this study calculates Pearson correlation coefficients among the dependent variables, independent variables, and control variables.

The correlation test results for Dependent Variable X_1_ are reported in Table 3 and can be summarized as follows. First, regarding the relationship between the dependent variable—attitudes toward Chinese culture—and the core independent variables, no significant correlation is found between “whether the respondent has visited the Chinese mainland” and attitudes toward Chinese culture. This indicates that direct experience of visiting the Chinese mainland alone does not exhibit a stable linear association with attitudes toward Chinese culture. In contrast, “the respondent’s perceived increase in cognition of the Chinese mainland” is significantly negatively correlated with attitudes toward Chinese culture, suggesting that as Taiwanese youths perceive greater informational gains about the Chinese mainland, their scores on the Chinese Cultural Attitude Index tend to decline. Second, with respect to the zero-order correlations among the independent variables, “whether the respondent has visited the Chinese mainland” and “the respondent’s perceived increase in cognition of the Chinese mainland” show a significant positive correlation, albeit with relatively weak strength. Overall, the correlations among the independent variables are low and do not pose a substantive risk of multicollinearity, indicating that they can be reasonably included in subsequent multivariate regression analyses.

**Table 3:** Zero-order correlation matrix of core variables for Dependent Variable Y_1_ (N = 920).

Variables	Y	X_1_	X_2_	Male	Age	Edu
Chinese Cultural Attitude Index (Y_1_)	1					
Contact Variable (X_1_)	–.042	1				
Cognition Variable (X_2_)	–.122^**^	.160^**^	1			
Male	.01	–.062	.164^**^	1		
Age	–.164^**^	.120^**^	.055	–.016	1	
Years of education	.006	.195^**^	.019	–.019	–.221^**^	1

Note: Entries represent simplified Pearson correlation coefficients. ***p < 0.001，**p < 0.01，*p < 0.05.

Third, regarding the control variables, gender does not exhibit a significant correlation with attitudes toward Chinese culture, whereas age shows a significant negative correlation, indicating that older respondents tend to score lower on the Chinese Cultural Attitude Index. No significant correlation is observed between years of education and attitudes toward Chinese culture. In addition, age and years of education are significantly negatively correlated, which is consistent with general demographic patterns.

The correlation analysis results for Dependent Variable Y_2_ are presented in Table 4 and can be summarized as follows. First, with regard to the relationship between the dependent variable—Taiwan’s Local Cultural Attitudes—and the independent variables, both Independent Variable X_1_ and Independent Variable X_2_ exhibit significant positive correlations with local cultural attitudes. This indicates that both “whether the respondent has visited the mainland” and “the respondent’s perceived increase in cognition of the Chinese mainland” are associated with local cultural attitudes among Taiwanese youth in the same directional manner. Second, the zero-order correlations between the independent variables are consistent with those reported in Model 1 and therefore are not reiterated here. Third, regarding the relationships involving the control variables, gender does not show a significant correlation with local cultural attitudes, whereas age and years of education each display weak positive correlations with local cultural attitudes. In addition, a significant negative correlation exists between age and years of education, which accords with general demographic patterns.

**Table 4:** Zero-order correlation matrix of core variables for Dependent Variable Y_2_ (N = 920).

Variables	Y	X_1_	X_2_	Male	Age	edu
Taiwan’s Local Cultural Attitude Index (Y_2_)	1					
Contact Variable (X_1_)	.089^**^	1				
Cognition Variable (X_2_)	.119^**^	.160^**^	1			
Male	–.053	–.062	.164^**^	1		
Age	.072^*^	.120^**^	.055	–.016	1	
Years of education	.080^*^	.195^**^	.019	–.019	–.221^**^	1

Note: Entries represent simplified Pearson correlation coefficients. ***p < 0.001，**p < 0.01，*p < 0.05.

It should be noted that correlation analysis reflects only bivariate linear associations between variables and does not control for the influence of other factors, nor does it allow for causal inference. Accordingly, the subsequent analysis employs multivariate regression models, controlling for demographic variables, to further examine the independent effects of direct contact and cognitive factors on cultural attitudes among Taiwanese youth.

### Empirical results

Prior to the empirical analysis, multicollinearity tests were conducted. To further assess potential multicollinearity in the models, this study evaluates both variance inflation factors (VIFs) and collinearity diagnostics. The results show that the condition indices for all explanatory variables do not exceed 30, and there is no instance in which multiple variables simultaneously exhibit high variance proportions within the same high condition index dimension. These findings indicate that the models do not suffer from serious multicollinearity problems.

**Fig 1:**
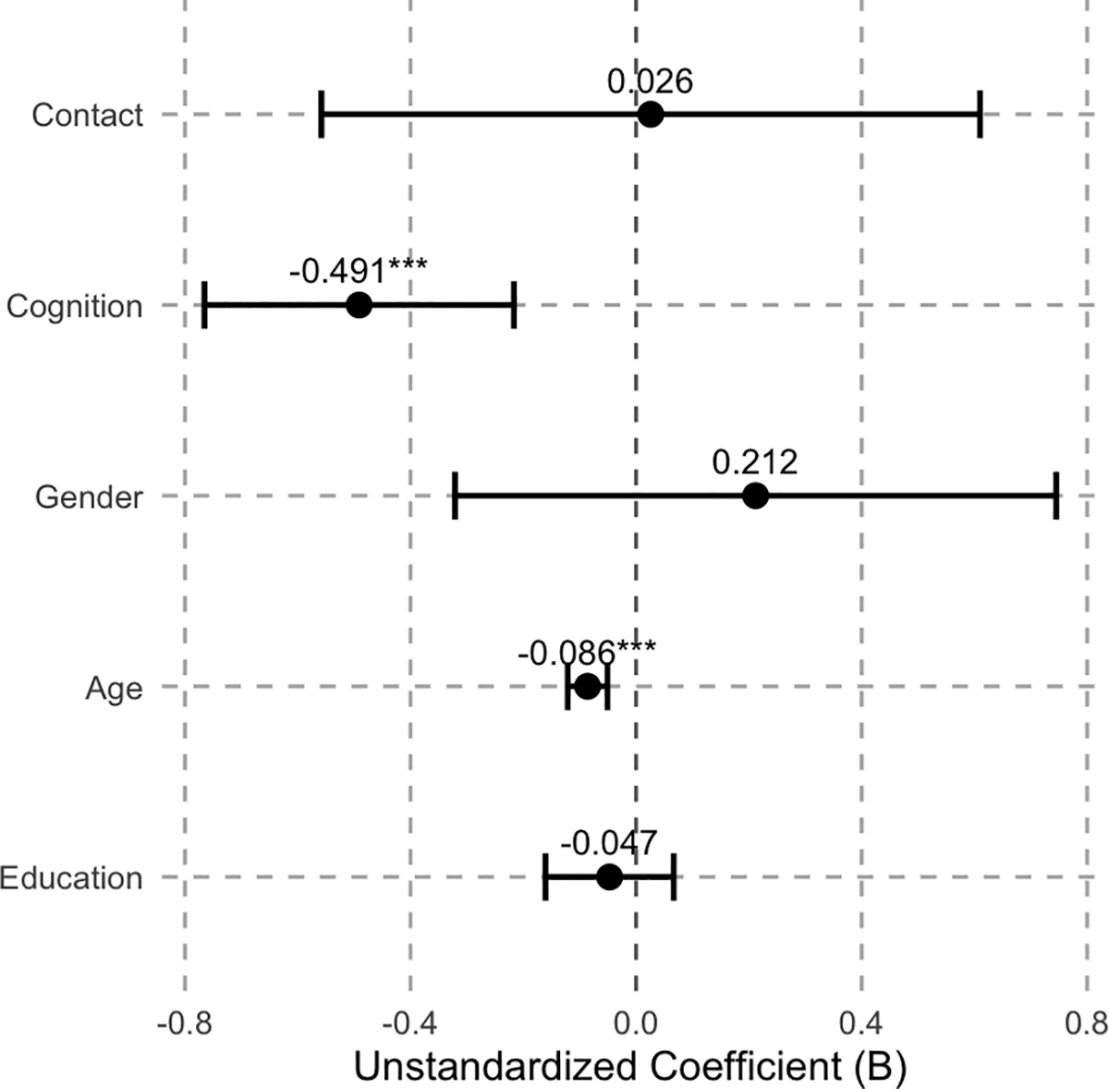
Coefficient Plot of OLS Regression Results for Model 1.

**Fig 2:**
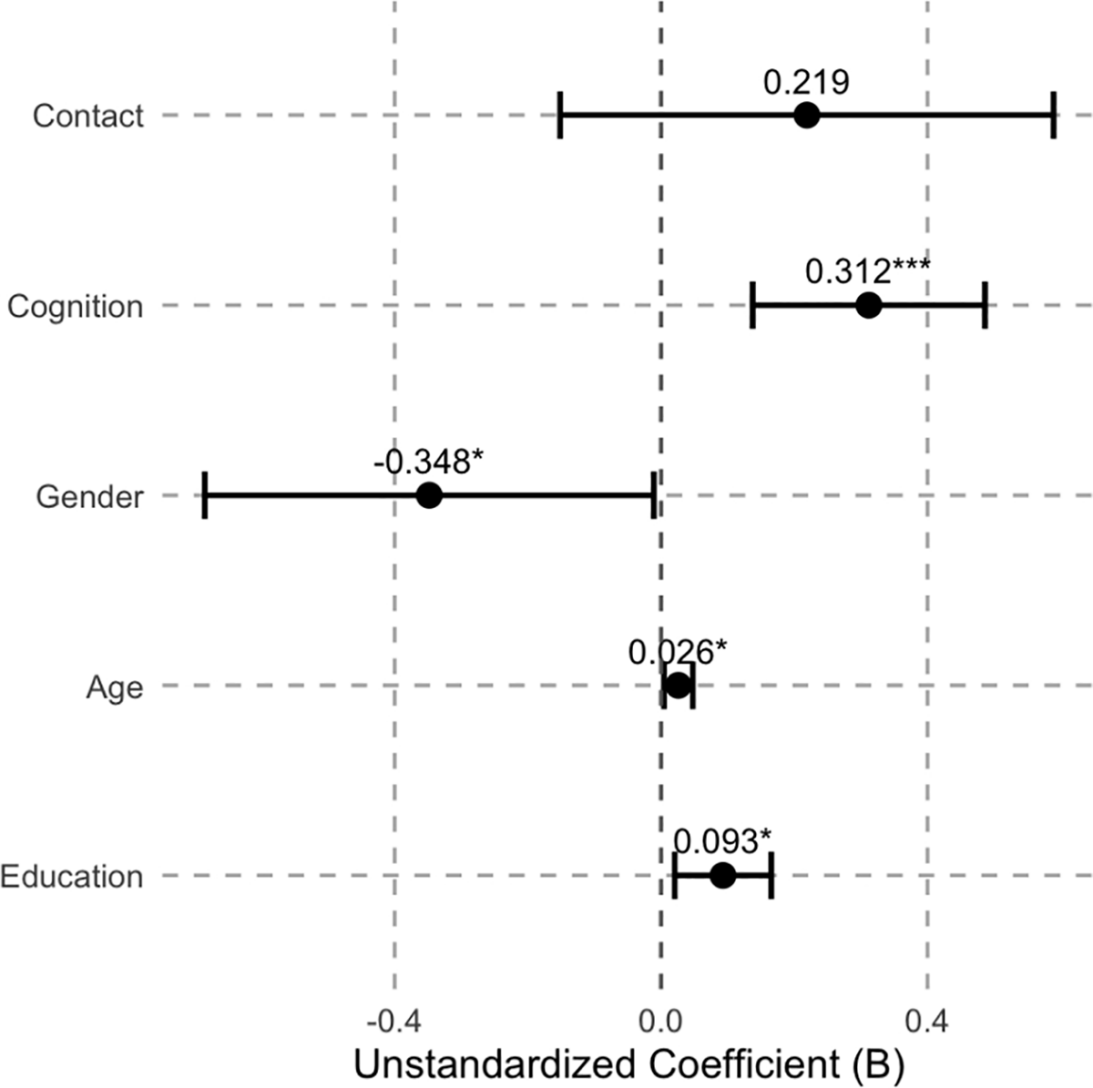
Coefficient Plot of OLS Regression Results for Model 2.

The regression results are presented in Table 5 and Figs 1 and 2. It should be emphasized that both cultural attitude indices are constructed by summing the original items according to their original coding directions, without reverse coding. Accordingly, lower scores indicate a higher level of respondents’ acceptance of the corresponding cultural orientation, whereas higher scores indicate a weaker degree of acceptance.

**Table 5:** Empirical Results of the Cultural Attitude Indices.

	Model 1: Chinese Cultural Attitudes	Model 2: Taiwan’s Local Cultural Attitudes
Constant	14.493***	7.505***
X_1_	0.026	0.219
X_2_	–0.491***	0.312***
Gender	0.212	–0.348*
Age	–0.086***	0.026*
Education	–0.047	0.093*
R²/ Adj. R²	0.032/ 0.027	0.032/0.027

Notes: Standard errors are reported in parentheses. *** p < 0.001, ** p < 0.01, * p < 0.05.

The main findings are as follows. In Model 1, which examines attitudes toward Chinese culture, the coefficient for the core independent variable whether the respondent has visited the Chinese mainland is not statistically significant. This indicates that, after controlling for demographic variables, personal contact experience per se is insufficient to produce a significant change in Taiwanese youths’ attitudes toward Chinese culture. By contrast, the respondent’s perceived increase in cognition of the Chinese mainland exerts a significant negative effect, implying that an increase in cognition significantly lowers the Chinese cultural attitude score—that is, it significantly strengthens Taiwanese youths’ acceptance of Chinese culture. With respect to the control variables, age has a significant negative effect on attitudes toward Chinese culture, suggesting that older respondents tend to have lower attitude scores and thus a higher level of acceptance of this culture. Gender and years of education do not reach statistical significance.

In Model 2, which examines attitudes toward local culture, the effect of whether the respondent has visited the Chinese mainland on local cultural attitudes is not statistically significant. This indicates that, after controlling for other factors, having direct contact experience with the Chinese mainland does not by itself significantly influence Taiwanese youths’ attitudes toward local culture. Second, the respondent’s perceived increase in cognition of the Chinese mainland has a significant positive effect on local cultural attitudes. An increase in cognition significantly raises the local cultural attitude score, meaning that as the respondent’s perceived understanding of the Chinese mainland increases, their acceptance of local cultural orientation relatively decreases. Regarding control variables, gender has a significant effect on local cultural attitudes, with male respondents scoring significantly lower than females. Additionally, both age and years of education show significant positive effects, indicating that as age and educational attainment increase, Taiwanese youths’ acceptance of local culture gradually declines.

Additionally, three points need to be noted. First, although the R^2^ values in both regressions are relatively low, this stems from the multifactorial nature of individual cultural attitude formation, which is also influenced by factors such as family upbringing and media exposure [48]. Second, this study is based on secondary survey data, and under current conditions, Taiwanese youths’ exchanges with the Chinese mainland are mostly short-term and episodic. This makes it difficult to more precisely quantify the cognitive changes induced by direct contact. Future research could build on this by incorporating panel data or experimental designs to differentiate the effects of various forms of contact on cognitive transformation, thereby systematically testing the potential mediating role of cognition between contact and cultural attitudes. Third, the results show that the effects of control variables differ across the two models, indicating that the social bases of different cultural attitudes are not entirely the same. In fact, Chinese cultural attitudes tend to reflect an intergenerationally transmitted cultural orientation, with changes primarily dependent on cognitive restructuring, whereas local cultural attitudes are more strongly shaped by contemporary social constructs and are more susceptible to factors such as gender roles, life-cycle stage (age), and educational experiences.

## Discussion

In both Model 1 and Model 2, the independent variable X_1_, “whether the respondent has visited the Chinese mainland” fails to reach statistical significance. This finding is broadly consistent with previous studies on the “limited effect of contact”. As noted in the literature review, whether through short-term visits to the Chinese mainland or long-term exposure to high-frequency economic and trade exchanges, mere contact alone is insufficient to produce significant changes in Taiwanese youths’ attitudes toward the Chinese mainland. This aligns with social identity theory, which suggests that in highly politicized societies, individuals tend to adopt defensive cognition to protect their identity, and are more likely to favor their in-group to maintain a sense of superiority [49,50]. This dynamic also relates to contact theory’s emphasis on the duration, mode, and frequency of contact. At present, cross-Strait youth exchanges are generally brief and shallow, and young people’s understanding of society in the Chinese mainland remains fragmented and superficial. Therefore, having visited the Chinese mainland does not necessarily imply the formation of a systematic understanding of its society and culture, nor does it readily trigger Taiwanese youths to reflect on their local cultural attitudes or shift toward Chinese culture. Taken together, the empirical results of this study and relevant literature indicate that, within the particular cross-Strait context, contact cannot be simply regarded as a sufficient condition for updating out-group cognition or altering cultural attitudes.

In both regression models, the core independent variable X_2_, “the respondent’s perceived increase in cognition of the Chinese mainland”, exhibits a significant effect, indicating that changes at the cognitive level better explain variations in Taiwanese youths’ cultural attitudes compared with X_1_, “whether the respondent has visited the Chinese mainland”. It should be emphasized that the cultural attitude indices in this study were constructed by summing the original item scores without reverse coding, so lower scores on the dependent variables indicate higher levels of cultural acceptance. Based on this coding logic, Model 1 shows that an increase in perceived cognition of the mainland has a significant negative effect on the Chinese Cultural Attitude Index. This means that as respondents perceive greater understanding of the mainland through online contact, their scores on the Chinese Cultural Attitude Index decrease, reflecting a significant increase in acceptance of Chinese culture.

Model 2 shows that the respondent’s perceived increase in cognition of the Chinese mainland has a significant positive effect on local cultural attitudes, meaning that the more respondents perceive an increase in their understanding of the Chinese mainland, the higher their Taiwan’s local cultural attitude scores, which corresponds to a relative decrease in acceptance of local cultural orientations. Together, the two results form a consistent pattern: cognitive increase does not merely affect a single cultural attitude but simultaneously drives a divergent change, enhancing acceptance of Chinese culture while weakening acceptance of local culture. This finding not only confirms the importance of increased out-group cognition emphasized in intergroup contact theory but also provides a particularly interesting case from Taiwan. Existing literature notes that contact theory highlights multiple mechanisms through which contact operates, including affective responses, perceived threat, intergroup anxiety, and cognitive changes [51]. But there is a lack of empirical cases under conditions of intense political confrontation.

At the same time, given current global developments, intergroup contact has expanded to include multiple forms, such as direct contact, electronic contact, imagined contact, vicarious contact, and quasi-social contact [52]. Taking the perceived increase in Taiwanese youths’ cognition of the Chinese mainland through online contact as an independent variable, the empirical analysis shows that the potential impact of contact on Taiwanese youths’ cultural attitudes does not primarily depend on whether contact occurs, but rather on whether the contact is accompanied by cognitive gains that can be absorbed and internalized by the individual, along with the meaning-making that arises from it. Furthermore, this study finds that the degree of cognitive change is a crucial entry point for explaining differences in the effects of contact. Further integrating Borinca’s research [53], the effect of contact depends on whether individuals complete cognitive processing and meaning-making of out-group information during the contact. Overall, under the current conditions in which cross-Strait interaction coexists with online contact, this study provides an empirical case demonstrating how “increased cognition” can simultaneously influence attitudes toward different cultural orientations.

Given that different cultural attitudes rest on distinct social foundations, the effects of control variables also exhibit differentiated patterns across the two models. Specifically, in the Taiwan’s local cultural attitude model, all three demographic variables display relatively clear and interpretable effects. Accordingly, this section focuses on discussing the results of the control variables in Model 2. Existing studies widely indicate that demographic characteristics exert systematic influences on the formation of cultural and political attitudes. With regard to gender, some survey research has found that Taiwanese female youths are less nationalist than their male counterparts—that is, they are less inclined to emphasize a Taiwan-centered identity [44]. Given that different cultural attitudes rest on distinct social foundations, the effects of control variables also exhibit differentiated patterns across the two models.

Specifically, in the local cultural attitude model, all three demographic variables display relatively clear and interpretable effects. Accordingly, this section focuses on discussing the results of the control variables in Model 2. Existing studies widely indicate that demographic characteristics exert systematic influences on the formation of cultural and political attitudes. With regard to gender, some survey research has found that Taiwanese female youths are less nationalist than their male counterparts—that is, they are less inclined to emphasize a Taiwan-centered identity [47]. The regression results of this study show that, after controlling for other factors, male respondents score significantly lower than female respondents on the Taiwan’s Local Cultural Attitude Index, indicating that males in this sample exhibit a stronger orientation toward local culture.

With respect to age, existing studies suggest that younger cohorts tend to display a more pronounced local identity orientation [46]. Consistent with this expectation, the present study finds that age has a significant positive effect on local cultural attitudes. That is, across different generations in Taiwan, younger respondents exhibit a higher level of acceptance of local culture, whereas older cohorts are relatively less inclined toward a local cultural orientation.

In addition, educational attainment, as an important variable in political socialization, should not be overlooked. Existing research suggests that specific nationalist narratives embedded in educational contexts may reinforce individuals’ awareness of identity boundaries [54]. The results of this study indicate that the longer the years of education, the weaker the respondents’ acceptance of local culture. This suggests that educational attainment in this sample does not directly correspond to a stronger local cultural orientation, but may instead reflect a more complex configuration of cognition and attitudes. After controlling for the aforementioned demographic factors, the core independent variables remain consistent with theoretical expectations in both direction and statistical significance, further demonstrating that the model structure is closely aligned with the research question and exhibits strong internal consistency and explanatory power.

By contrast, in the Chinese cultural attitude model, neither gender nor years of education reach statistical significance, while the direction of the age effect remains consistent across the two models. The non-significant role of gender in shaping attitudes toward Chinese culture may be attributable to the long-term institutional marginalization and de-contemporization of Chinese culture in education and public discourse, whereby identification with Chinese culture is largely confined to an abstract cultural level. Under such conditions, potential gender differences in cultural attitudes are substantially compressed. Similarly, the finding that longer years of education do not significantly increase acceptance of Chinese culture may reflect a more complex mechanism. Although individuals with higher levels of education are more exposed to cross-cultural and globalized information and may adopt relatively rational or critical attitudes toward local culture—particularly toward the version of Taiwanese local culture constructed by the Democratic Progressive Party—this does not necessarily translate into a stronger inclination toward Chinese culture. In other words, even when the two cultural orientations are positioned as mutually exclusive in public discourse, rejecting one does not automatically imply embracing the other, much as some Taiwanese citizens may dislike the Democratic Progressive Party without favoring the Chinese Kuomintang.

It should be noted that this study has certain limitations, as the data only reflect the attitudinal structure of the youth group within the cross-Strait and Taiwanese political-social context in 2018. After 2018, due to the COVID-19 pandemic and deliberate restrictions imposed by the Democratic Progressive Party, cross-Strait exchanges gradually diminished and did not begin to recover until 2023. On the one hand, since 2018, cross-Strait relations have become increasingly tense, and Taiwanese public threat perception of the Chinese mainland has intensified, which may affect national identity and further incline cultural attitudes toward local culture. On the other hand, in recent years, the rapid development of Chinese mainland social media has led to a sharp increase in Taiwanese youth users of platforms such as TikTok and Red Note App. During this period, virtual contact may enhance individuals’ cognition of the Chinese mainland, and the positive effects of such cognition may be amplified in reality. Future studies may consider conducting online surveys to assess Taiwanese youth’s initial level of ethnic identity, as well as their contact experiences with the Chinese mainland, including contact duration and intensity. Subsequently, researchers could further measure participants’ cognitive understanding of the Chinese mainland, in order to more rigorously examine the underlying causal mechanisms.

In sum, the empirical results of this study further demonstrate that, in the context of cross-Strait interactions, contact per se is insufficient to automatically induce changes in Taiwanese youths’ cultural attitudes. What matters is not whether contact occurs, but whether such contact facilitates the accumulation of cognition about the Chinese mainland. Compared with contact experience alone, cognitive transformation is more likely to constitute a key mechanism shaping changes in Taiwanese youths’ local cultural attitudes.

## Conclusion

In sum, drawing on Taiwan Social Change Survey data from 2018 and within the analytical framework of intergroup contact theory, this study examines the statistical associations between Taiwanese youths’ whether the respondent has visited the Chinese mainland and the respondent’s perceived increase in cognition of the Chinese mainland, and their cultural attitudes. The findings indicate that, under conditions of heightened cross-Strait political tension and constrained face-to-face exchanges, having visited the Chinese mainland per se is not significantly associated with Taiwanese youths’ cultural attitudes. By contrast, increases in Taiwanese youths’ cognition of the Chinese mainland through online media are significantly related to their cultural attitudes. This suggests that under highly restricted contact conditions, direct contact experiences do not necessarily lead to changes in cultural attitudes; rather, it is the contact-induced positive cognitive transformation that is more likely to bring about such attitudinal change.

From a theoretical perspective, the findings of this study extend the applicability of intergroup contact theory to highly politicized and mediatized contexts. Existing research has largely focused on the role of direct contact in reducing prejudice and improving intergroup attitudes. This study shows that under conditions in which real-world contact is constrained and the information environment is highly fragmented, internet-based indirect contact and the cognitive changes associated with it may exert a meaningful influence on cultural attitudes.

From a methodological perspective, this study relies on cross-sectional survey data and therefore primarily identifies associations between variables rather than making causal claims. Due to data limitations, the indicator of “cognitive change” used in this study captures respondents’ subjectively perceived ranking of information exposure to the Chinese mainland, rather than their level of objective knowledge about Chinese mainland society. Moreover, the possibility of self-selection or reverse causality cannot be fully ruled out.

Overall, this study empirically demonstrates that, against the backdrop of changing cross-Strait interaction structures, understanding the formation of Taiwanese youths’ cultural attitudes requires attention to both forms of contact and individual-level cognitive processes. The findings provide exploratory empirical evidence for further discussions on the scope of applicability of intergroup contact theory in complex political and social contexts, as well as on the multiple conditions underlying the formation of youth cultural attitudes.

## Supporting information

S1 Data Data. (ZIP)
